# Hypoxia-induced PLOD2 promotes proliferation, migration and invasion via PI3K/Akt signaling in glioma

**DOI:** 10.18632/oncotarget.16710

**Published:** 2017-03-30

**Authors:** Ye Song, Shihao Zheng, Jizhou Wang, Hao Long, Luxiong Fang, Gang Wang, Zhiyong Li, Tianshi Que, Yi Liu, Yilei Li, Xi’an Zhang, Weiyi Fang, Songtao Qi

**Affiliations:** ^1^ Department of Neurosurgery, Nanfang Hospital, Southern Medical University, Guangzhou, Guangdong, 510515, PR China; ^2^ Department of Pharmacology, Nanfang Hospital, Southern Medical University, Guangzhou, Guangdong, 510515, PR China; ^3^ Cancer Center, TCM-Integrated Hospital, Southern Medical University Guangzhou, Guangdong, 510515, PR China

**Keywords:** PLOD2, glioma, epithelial–mesenchymal transition, PI3K/AKT, hypoxia

## Abstract

Gliomas are the most common form of malignant primary brain tumors with poor 5-year survival rate. Dysregulation of procollagen-lysine, 2-oxoglutarate 5-dioxygenase 2 (PLOD2) was observed in gliomas, but the specific role and molecular mechanism of PLOD2 in glioma have not been reported yet. In this study, PLOD2 was found to be frequently up-regulated in glioma and could serve as an independent prognostic marker to identify patients with poor clinical outcome. Knockdown of PLOD2 inhibited proliferation, migration and invasion of glioma cells *in vitro* and *in vivo*. Mechanistically, inhibition of PLOD2 inactivated PI3K/AKT signaling pathway and thus regulated the expression of its downstream epithelial–mesenchymal transition (EMT)-associated regulators, including E-cadherin, vimentin, N-cadherin, β-catenin, snail and slug in glioma cells. Moreover, PLOD2 could be induced by hypoxia-inducible factor-1α (HIF-1α) via hypoxia, thereby promoting hypoxia-induced EMT in glioma cells. Our data suggests that PLOD2 may be a potential therapeutic target for patients with glioma.

## INTRODUCTION

Gliomas are the most common form of malignant primary brain tumors in adults, with an annual incidence of approximately four to five per 100,000 people [1]. Despite of the therapeutic advances that had been made in the past few decades, such as surgical resection, adjuvant radiotherapy and chemotherapy, the overall 5-year survival rate of glioma patients still remains poor [2, 3]. Particularly, majority of glioma patients succumb to this disease within 2 years of diagnosis [4]. Thus, a great challenge lies ahead in understanding the molecular mechanisms of glioma tumorigenesis to identify novel prognostic molecular markers as well as to develop novel therapeutic strategies.

Procollagen-lysine, 2-oxoglutarate 5-dioxygenase 2(PLOD2), also known as LH2, TLH2 or BRKS2, is a membrane-bound homodimeric enzyme that specifically hydroxylates lysines in the telopeptide of procollagens [5]. A collection of studies has demonstrated that PLOD2 is essential for the biogenesis of normal mature collagen and the stability of collagen cross-links [6, 7]. Due to the crucial role of collagen in formation of extracellular matrix, PLOD2 has been implicated in various pathological processes, including systemic sclerosis [8], osteogenesis imperfecta [9] and join contractures [10]. Importantly, deregulation of PLOD2 has been observed in several kinds of cancers [11–13]. PLOD2 expression has been found to provide prognostic information about bladder cancer, hepatocellular carcinoma and glioblastoma [13–15]. Additionally, PLOD2 was detected to be involved in agiogenesis after ischemic stroke, specifically related to reconstruction of basal lumina, this correlation implies that PLOD2 might be a potential target in anti-angiogenesis treatment [16].

Hypoxia is a critical aspect of the tumor microenvironment [17]. Accumulating evidence supports important associations between hypoxia and tumorigenesis, such as poor prognosis, increased angiogenesis, tumor growth and resistance to radio-and chemotherapy [18]. Hypoxia-inducible factor-1α (HIF-1α), the key hypoxia regulatory factor, could translocate to the nucleus under hypoxia and then induce the transcription of numerous downstream target genes by binding to hypoxia response elements (HREs), thereby participating in diverse biological processes during tumor development and progression [19, 20]. Interestingly, previous studies have shown that PLOD2 can be induced by HIF-1α under hypoxia condition [21, 22]. However, whether HIF-1α increases PLOD2 expression by directly binding to HREs or whether PLOD2 is involved in hypoxia-mediated glioma tumorigenesis still remains unclear.

In this study, we characterized the biological roles of PLOD2 in glioma tumorigenesis and investigated the underlying mechanisms. Our investigation provides evidence that inhibition of PLOD2 attenuates glioma cell migration and invasion both *in vitro* and *in vivo* via the modulation of PI3K/AKT signaling pathway and promotion of EMT. Interestingly, our results reveal that PLOD2 is directly induced by HIF-1α through the HREs located within the PLOD2 promoter and therefore may mediate hypoxia-induced EMT in glioma cells. Taken together, these data demonstrate that hypoxia-induced PLOD2 promotes EMT via PI3K/Akt signaling in glioma.

## RESULTS

### PLOD2 is frequently up-regulated in glioma

**Figure 1:**
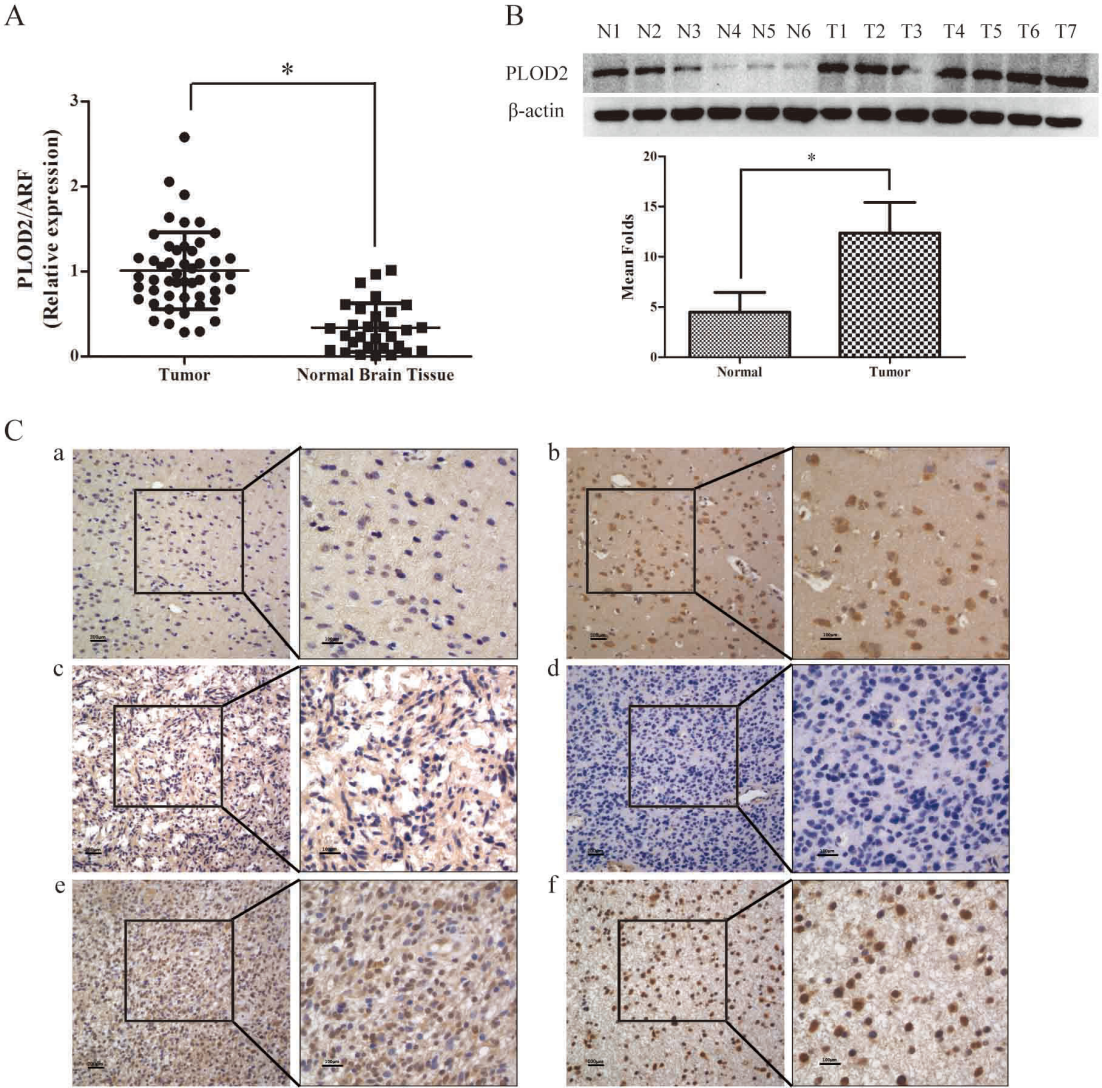
PLOD2 expression in glioma and normal brain tissues (**A**) The expression level of PLOD2 mRNA was decreased in normal brain tissues compared with glioma tissues by RT-PCR. Data were presented at mean ± SD for three independent experiments (*P < 0.05). (**B**) The expression level of PLOD2 protein was evaluated in 7 glioma tissue samples compared with 6 normal brain tissue samples by western blot. The unpaired *t* test was used for this assay (*P < 0.05). (**C**) PLOD2 expression in glioma and normal brain tissues was examined by immunohistochemical staining. a).weak staining of PLOD2 in normal tissues. b).strong staining of PLOD2 in normal tissues. c) and d). weak staining of PLOD2 in glioma tissues. e) and f). strong staining of PLOD2 in glioma tissues. Original magnification 400×

To determine whether PLOD2 plays a role in glioma tumorigenesis, quantitative real time PCR (qRT-PCR) was initially conducted to determine the expression of PLOD2 mRNA transcripts in 50 frozen glioma tissues and 30 normal brain tissues. As shown in Figure 1A, the glioma tissues showed markedly overexpression of PLOD2 (*P* < 0.001). Up-regulation of PLOD2 protein was confirmed in 7 tumor samples and 6 normal samples by western blot analysis (*P* < 0.001) (Figure 1B). Furthermore, immunohistochemical staining of PLOD2 protein was performed with 125 paraffin-embedded glioma samples and 30 normal brain tissues. High protein level was found in 59.2% (74 of 125) of glioma tissues, compared with only 36.67% (11 of 30) of normal tissues (*P* = 0.026) (Figure 1C, Table 1).

**Table 1:** Protein expression of PLOD2 between glioma and NB tissues

Group	Cases	Protein expression		*P* value
		High expression	Low expression	
Glioma	125	74	51	
Normal	30	11	19	0.026

### PLOD2 knockdown attenuates glioma cell proliferation, migration and invation *in vitro*

To determine the biological functions of PLOD2, the expression levels of PLOD2 inseveral glioma cell lines were analyzed by western blot. Five glioma cell lines, including U251, U87, U118MG, A172 and SHG44, exhibited different levels of PLOD2 expression (Supplementary Figure 1). Given the fact that U87 and U251 have relatively higher endogenous PLOD2 expression, we chose these 2 cell lines for PLOD2 silencing experiments.

**Figure 2:**
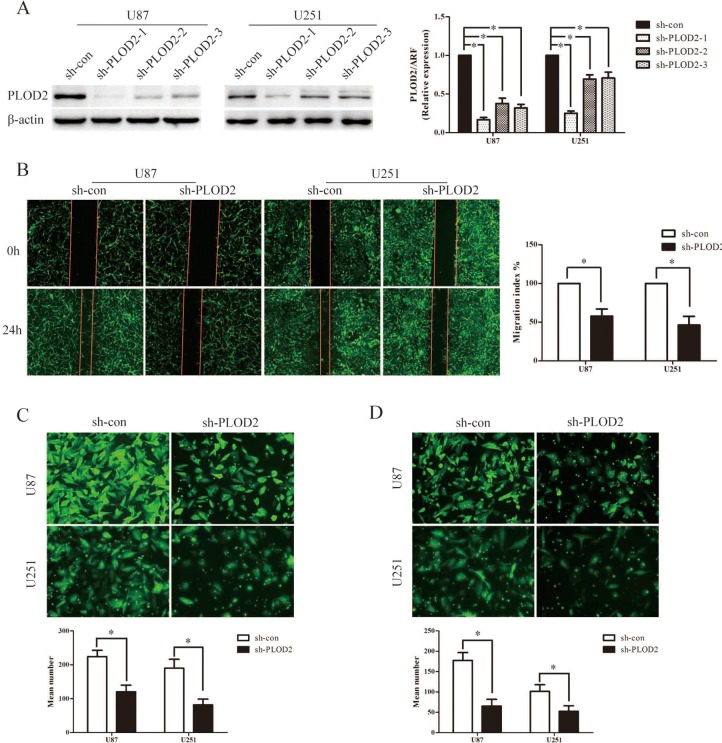
Stable suppression of PLOD2 attenuates the migration and invasion ability in glioma cells *in vitro* (**A**) U251 and U87 cells were infected with three short hairpin RNA vectors against PLOD2, and U251 and U87 cells with stably down-regulated PLOD2 expression were screened by western blot. β-actin served as a loading control. Bar graph shows the relative expression of protein among the groups. Data are presented as mean ± SD for three independent experiments (*P < 0.05). (**B**) Wound healing assay showed U251 and U87 cells with sh-PLOD2 or sh-con vectors at 0 and 24 h after wounding. Bar chart showed the relative migration ability at 24 h. (**C**) Transwell chamber assays indicated that stably down-regulated PLOD2 reduced the migration ability of glioma cells *in vitro*. (**D**) Boyden chamber assays revealed that stably suppressed PLOD2 expression inhibited invasiveness of glioma cells *in vitro*. All values shown are mean ± SD of triplicate measurements and have been repeated 3 times with similar results (*P < 0.05). Independent *t* test was used to determine the differences between two groups.

**Figure 3:**
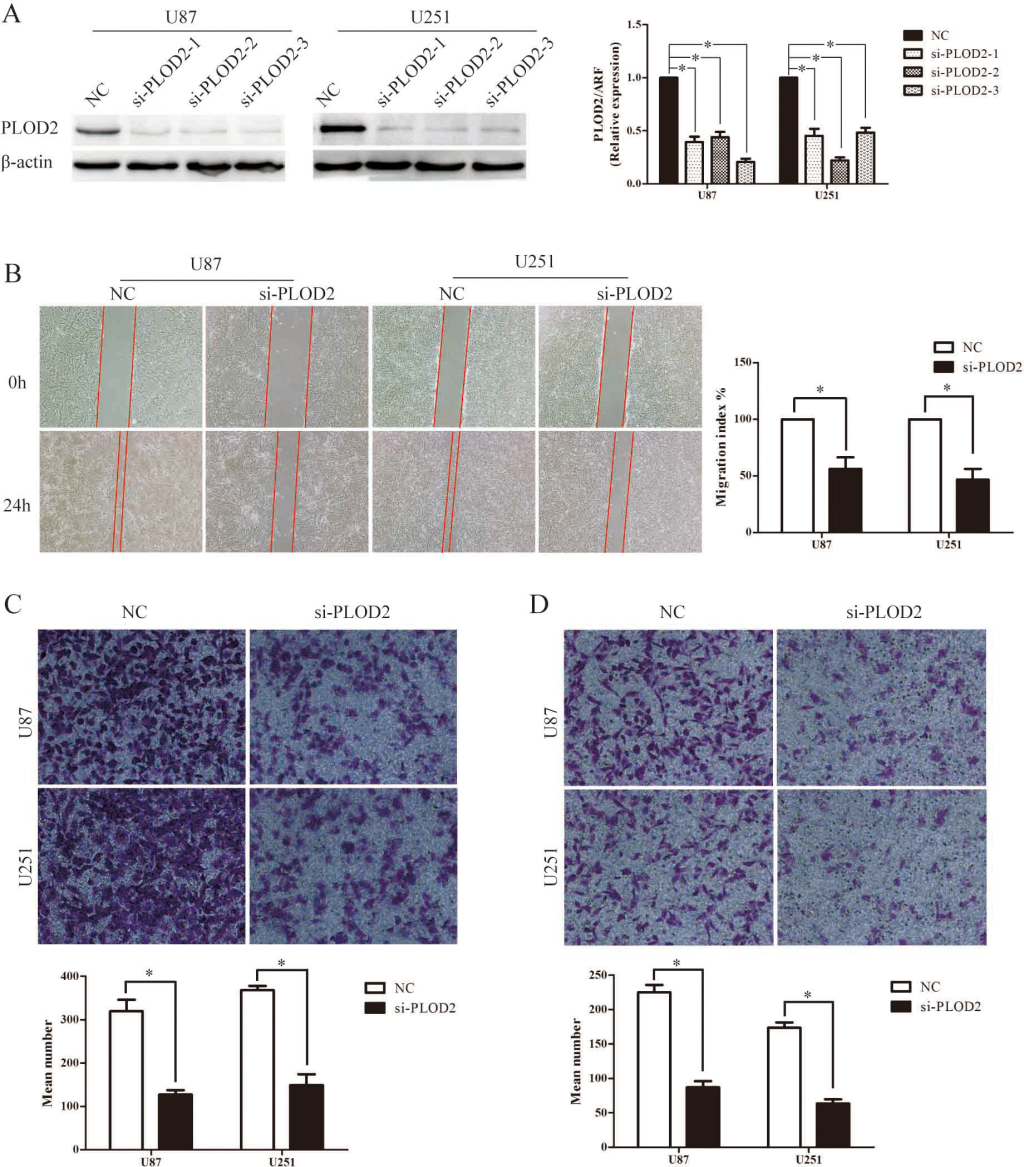
Transient depletion of PLOD2 reduces cell migration and invasion ability *in vitro* (**A**) Three siRNAs against PLOD2 decreased the expression of PLOD2 in U251 and U87 cells compared with control cells by western blot. β-actin was used as an internal control. Bar graph showed the relative expression of protein among the groups. Data were presented as mean ± SD for at least three independent experiments (*P < 0.05). (**B**) Wound healing assay indicated that siPLOD2 transfection into U251 and U87 cells for 24 h impaired cell migrating capacity, compared with the negative control group. Bar chart showed the relative migration ability at 24 h. (**C**) Transwell chamber assays showed that transiently down-regulated PLOD2 reduced the migration ability of U251 and U87 cells *in vitro*. (**D**) Boyden chamber assays revealed that transiently decreased PLOD2 expression inhibited invasiveness of U251 and U87 cells *in vitro*. All values shown are mean ± SD of triplicate measurements and have been repeated 3 times with similar results (*P < 0.05). Independent *t* test was used to determine the differences between two groups.

Subsequently, three lentiviral shRNA vectors were used to specifically and stably knock down the expression of PLOD2 in U251 and U87 cells. The efficiency of the three shRNA candidates was confirmed by qRT–PCR and western blot analysis (Figure 2A). Among them, the most efficient shRNA vector, sh-PLOD2-1, was selected for further analysis. MTT assays were performed to determine its fucnction on cell proliferation. After stably transfected sh-PLOD2-1, both U251 and u87 showed a slower rate of proliferation compared to the control group (Supplementary Figure 2A). However, the proliferation rate did not show much difference after transiently silencing PLOD2 by siRNA within three days observation (data not shown). To determine the function of PLOD2 in glioma migration and invasion, wound-healing assay, the transwell chamber and Boyden chamber assays were performed with U87 and U251 cells. In wound-healing assays, quantitative analysis at 48 h showed a significant reduction in wound closure in cells with silenced PLOD2 expression compared with control cells (Figure 2B). Similarly, in transwell chamber assays, knockdown of PLOD2 significantly decreased the percentage of migrated cells in the sh-PLOD2-transfected group (Figure 2C). Furthermore, Boyden chamber assays revealed that PLOD2 depletion significantly decreased the number of invaded cells in both U87 and U251 cells (Figure 2D). These data demonstrated that inhibition of PLOD2 suppressed glioma cell migration and invasion *in vitro*. Consistently, suppressing PLOD2 expression transiently with siRNA also significantly reduced cell migration and invasion in both U87 and U251 cells (Figure 3A–3D).

### PLOD2 modulates multiple EMT-associated factors: inactivation of PI3K/Akt signal in glioma cells was involved

**Figure 4:**
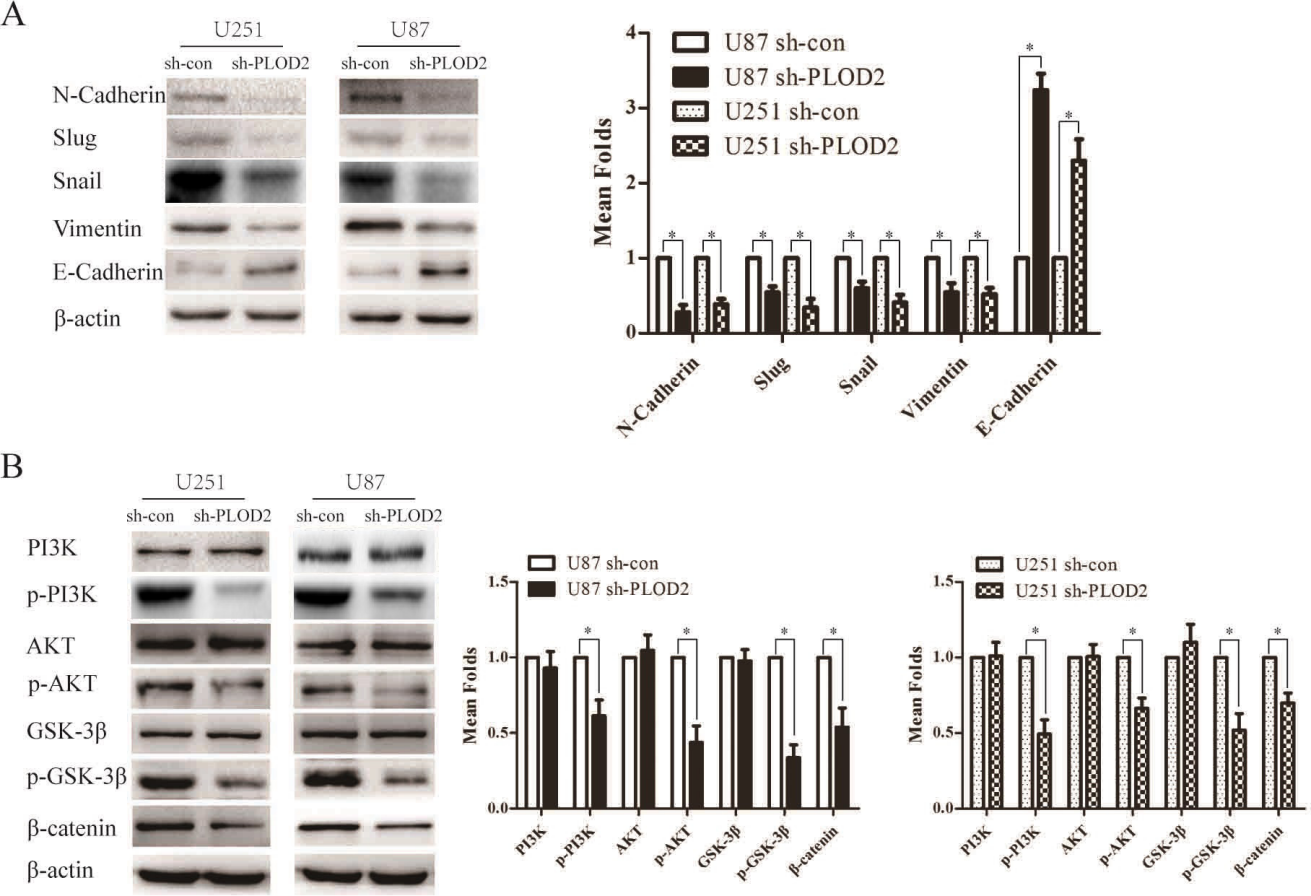
PLOD2 regulates the expression of EMT-associated genes in glioma cells through PI3K/AKT signaling pathways (**A**) Knockdown of endogenous PLOD2 in U251 and U87 cells reduced the expression of several EMT-marker genes including Snail, Slug, Vimentin and N-cadherin but enhanced E-cadherin expression. (**B**) Reduced PLOD2 expression significantly decreased the expression of phosphorylated PI3K, AKT, GSK3β and β-catenin, whereas total levels remained unchanged. β-actin was used as a loading control.

To obtain further insight into the mechanisms of PLOD2 in glioma cell migration and invasion, the expression levels of some of the EMT-associated regulators were examined using western blot analysis in U87 and U251cells with stably suppressed PLOD2 expression. Significant increases in the level of E-cadherin and decreases in the level of N-cadherin, slug, snail and vimentin were shown in PLOD2 knockdown U87 and U251 cells (Figure 4A). Moreover, knockdown of PLOD2 in U87 and U251cells resulted in decreased level of phosphorylated PI3K, AKT and GSK-3β whereas total levels of these proteins remained unchanged (Figure 4B). Additionally, the level of β-catenin was down-regulated in U87 and U251cells with silenced PLOD2 (Figure 4B). These results indicated that PLOD2 promotes EMT and is an upstream factor modulating the PI3K/Akt signaling pathways in glioma cells.

### Suppression of PLOD2 impaired the proliferative ability of glioma cells *in vivo*

Subcutaneous injection of shPLOD2 or sh-con U87 cells was performed and the tumor masses were harvested after 5 weeks. The volume of the tumor mass of the shPLOD2 group is significantly smaller than that of the sh-con group (Supplementary Figure 2B and 2C). This result support that knockdown of PLOD2 is detrimental for the proliferation of glioma cells *in vivo*.

### Knockdown of PLOD2 attenuates the migration and invasive potential of glioma cells *in vivo*

**Figure 5:**
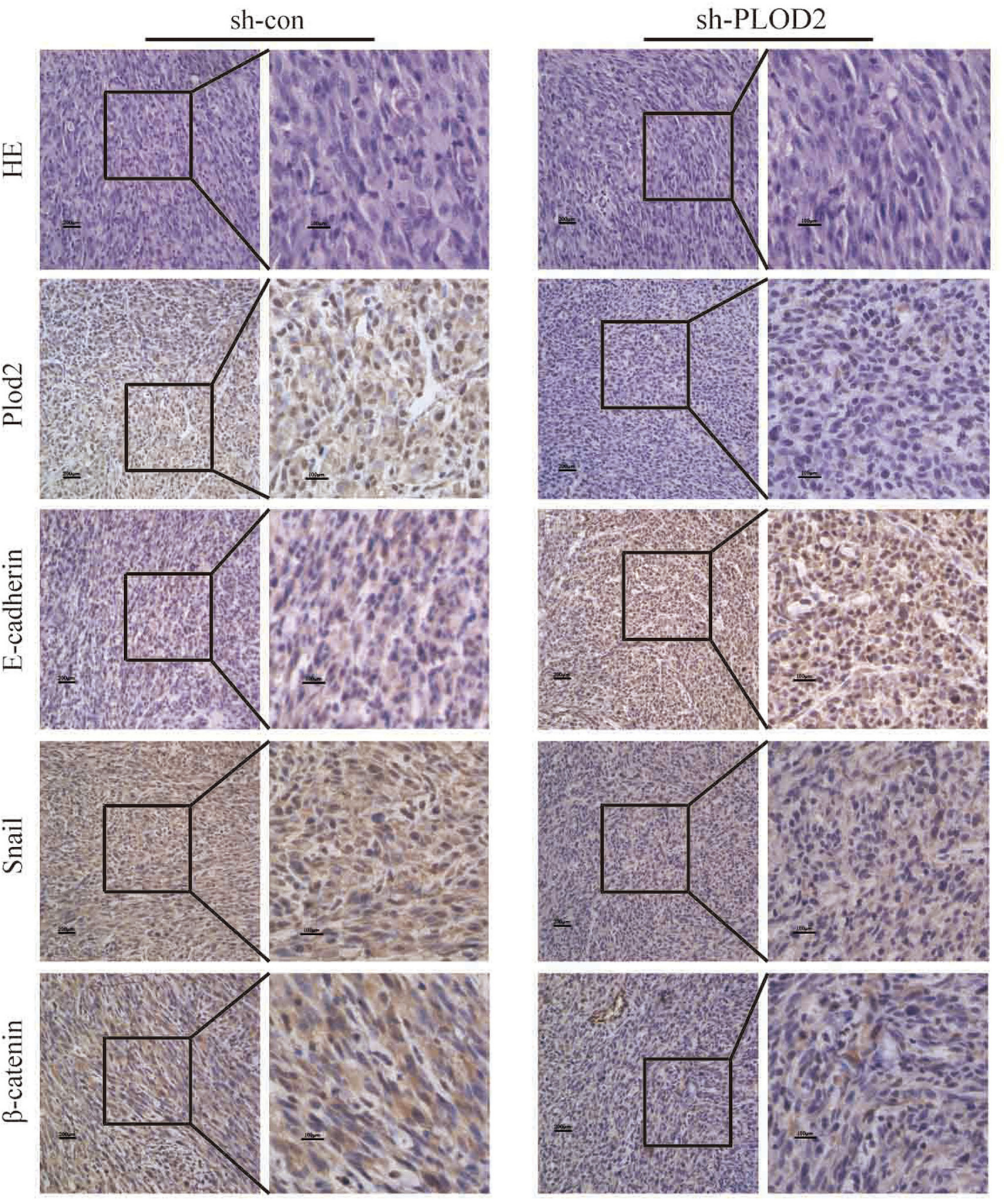
The expression of PLOD2, E-cadherin, Snail, and β-catenin in xenograft tumor of mice injected subcutaneously with sh-PLOD2 or sh-con U87 cells was detected by IHC

In order to reveal whether lower expression of PLOD2 has beneficial effects *in vivo*, we performed intracranial xenograft assay in nude mice. As shown in Supplementary Figure 3, most of the cells in the central of the tumor were replaced by stromal cells with only a few glioma cells surrounding the edge of the tract of injection. While in the control group, tumor cells survived in the center. On the other hand, fewer glioma cells migrated away from the injection tract in the shPLOD2 group than that in the control group. This might be explained by the theory that co-activation of PLOD2 and HIF-α is essential for tumor cell invasion and migration [21]. When PLOD2 is inactivated, the ability of glioma cells to survive hypoxia condition might be weakened. We have also performed intraspleen injection of glioma cells in nude mice. In this model, we found that in the metastasis nodule in the liver, control tumors showed extensive invasion into the adjacent tissue, whereas PLOD2-knockdown tumors maintained a distinct tumor–stroma boundary (Supplementary Figure 4). Further immunohistochemistry analysis of the tumor tissues from the subcutaneous xenografts revealed that the levels of PLOD2, snail and β-catenin were significantly lower in xenografts of sh-PLOD2 cells than in xenografts of sh-con cells while E-cadherin was markedly upregulated in xenografts of sh-PLOD2 cells, consistent with our *in vitro* findings (Figure 5). These results indicate that knockdown of PLOD2 inhibited the invasive potential of glioma cells and is an adverse factor for the survival of tumor cells *in vivo*.

### PLOD2 expression is induced by HIF-1α under hypoxia in glioma cells

**Figure 6:**
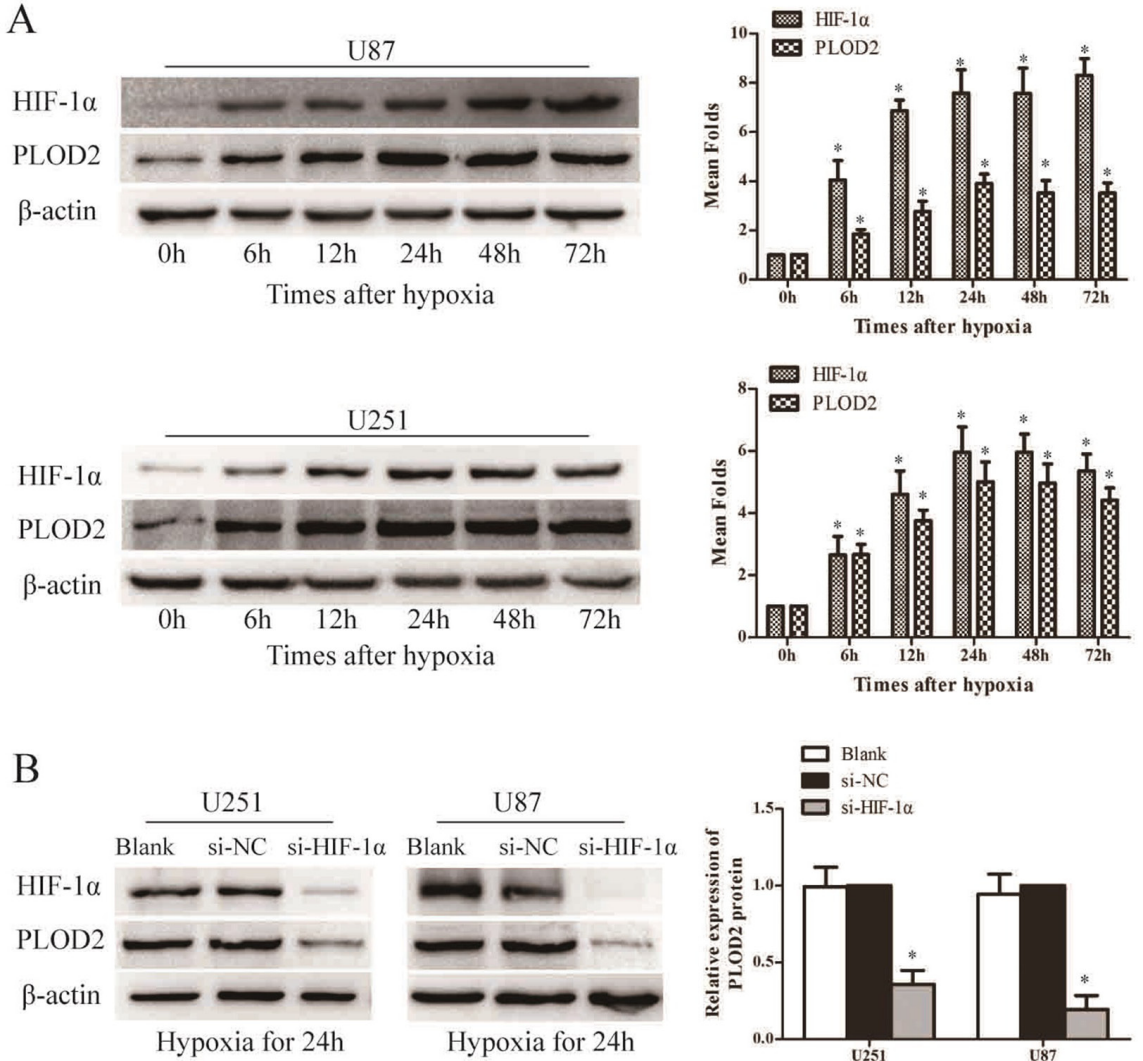
PLOD2 expression is directly regulated by HIF-1α (**A**) Western blot analysis of HIF-1α and PLOD2 protein expression after different times of hypoxia in U251 and U87 cells. (**B**) Western blot analysis of HIF-1α and PLOD2 protein expression in si- HIF-1α, si-NC and Blank U251 and U87 cells under low oxygen (*P < 0.05, compared to time 0 hr).

To ascertain the association between PLOD2 and hypoxia, U87 and U251 cells were cultured under hypoxic conditions for 6 h, 12 h, 24 h, 48 h and 72 h and the expression of PLOD2 was assessed by western blot analysis. As shown in Figure 6A, the expression level of PLOD2 protein in both U87 and U251 cells gradually increased under hypoxia after 6 h of exposure, reached a peak at 24 h, and was sustained at least to 72 h, consistent with the increase in HIF-1α protein level.

To explore whether hypoxia-induced PLOD2 is mediated by HIF-1α, U87 and U251 cells were transiently transfected with siRNAs against HIF-1α under hypoxic conditions. siRNAs significantly decreased HIF-1α protein levels in both U87 and U251 cells (Figure 6B). Importantly, PLOD2 protein was significantly reduced in cells transfected with HIF-1α siRNAs compared to those transfected with a scrambled siRNA, indicating that HIF-1α might increase PLOD2 expression (Figure 6B).

### PLOD2 mediates hypoxia-induced EMT in glioma cells

**Figure 7:**
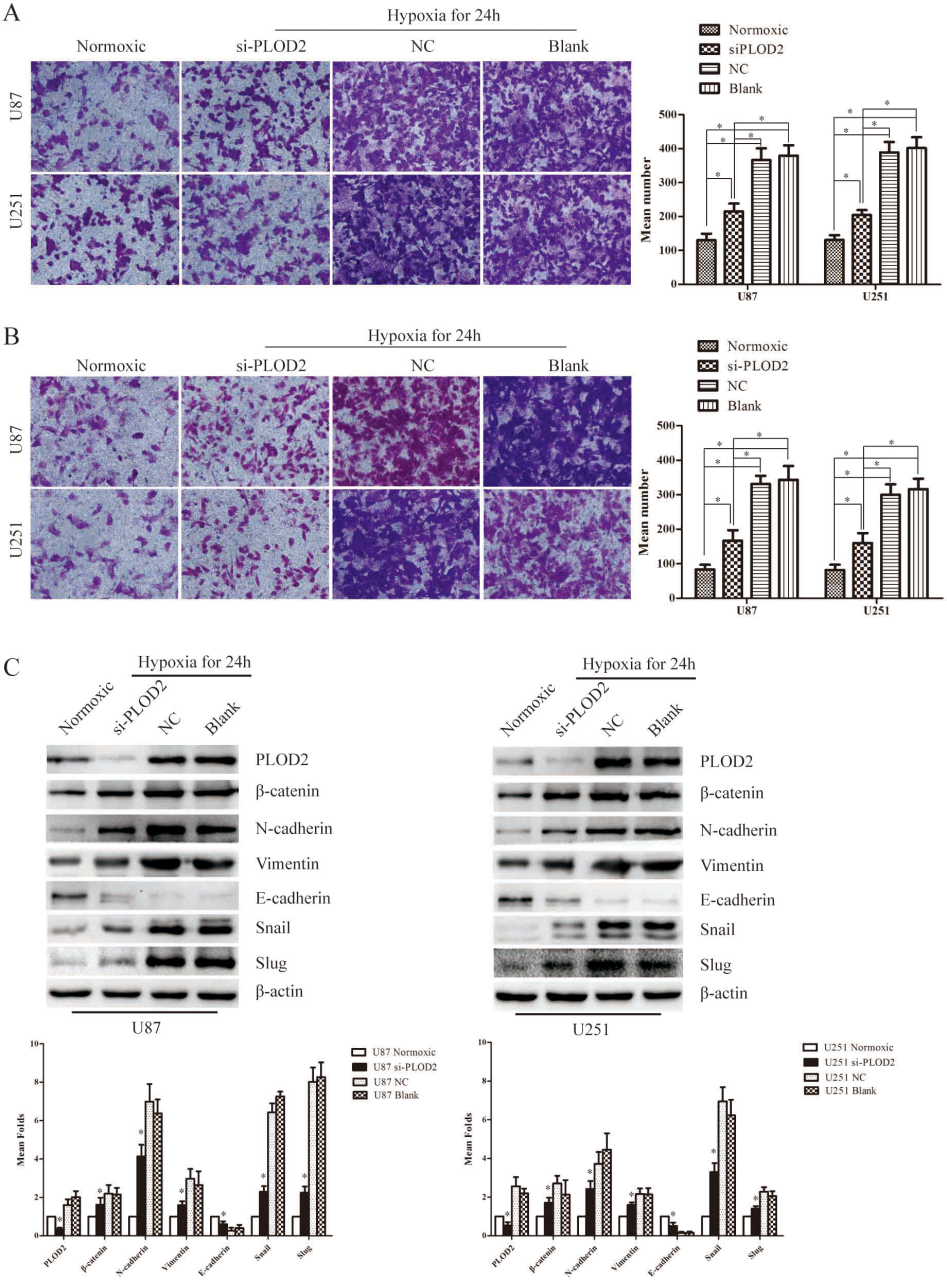
PLOD2 mediates hypoxia-induced EMT in glioma cells (**A**) Transwell chamber assays showed that hypoxia could significantly induced U251 and U87 cell migration and transiently down-regulated PLOD2 partly reversed the migration ability of U251 and U87 cells induced by hypoxia. The bar graph in the right panel represented the number of migrated cells in different groups. (**B**) Bodyen chamber assays showed that hypoxia could significantly induce U251 and U87 cell migration and transiently down-regulated PLOD2 partly reversed the migration ability of U251 and U87 cells induced by hypoxia. The bar graph in the right panel represented the number of invaded cells in different groups. (**C**) The expression levels of several EMT-associated genes including Snail, Slug, Vimentin, N-cadherin, E-cadherin and β-catenin were detected in U87 and U251 cells transfected with siPLOD2 after 24 h under hypoxia or normoxic state by western blot. β-actin was used as an internal control. Bar graph represented the relative expression of protein among the groups. Data shown are the mean ± SD of three independent experiments (*P < 0.05).

As described above, knockdown of PLOD2 modulated multiple EMT-associated proteins and thus blocked glioma cell migration and invasion both *in vitro* and *in vivo*. We then asked whether PLOD2 was involved in hypoxia-induced migration and invation in U87 and U251 cells. U87 and U251 cells were transfected with siRNAs against PLOD2 after 24 h under hypoxia or normoxic state. After another 48 h, cells were collected and seeded in Transwell chambers or Boyden chambers coated with matrigel. Indeed, PLOD2 depletion in U87 and U251 cells reversed cell migration and invasion induced by hypoxia (Figure 7A–7B). Additionally, we found that, compared with normoxic cells, E-cadherin was significantly down-regulated whereas β-catenin, N-cadherin, vimentin, snail and slug were up-regulated in hypoxic cells after 24 hours hypoxia treatment (Figure 7C). Transfection with siRNAs against PLOD2 reversed the decrease in E-cadherin levels and increase in β-catenin, N-cadherin, vimentin, snail and slug level in hypoxic cells to levels comparable with those in normoxic cells (Figure 7C). Taken together, PLOD2 mediated hypoxia-induced EMT in glioma cells.

### Increased expression of PLOD2 is unfavorable for glioma prognosis

**Figure 8:**
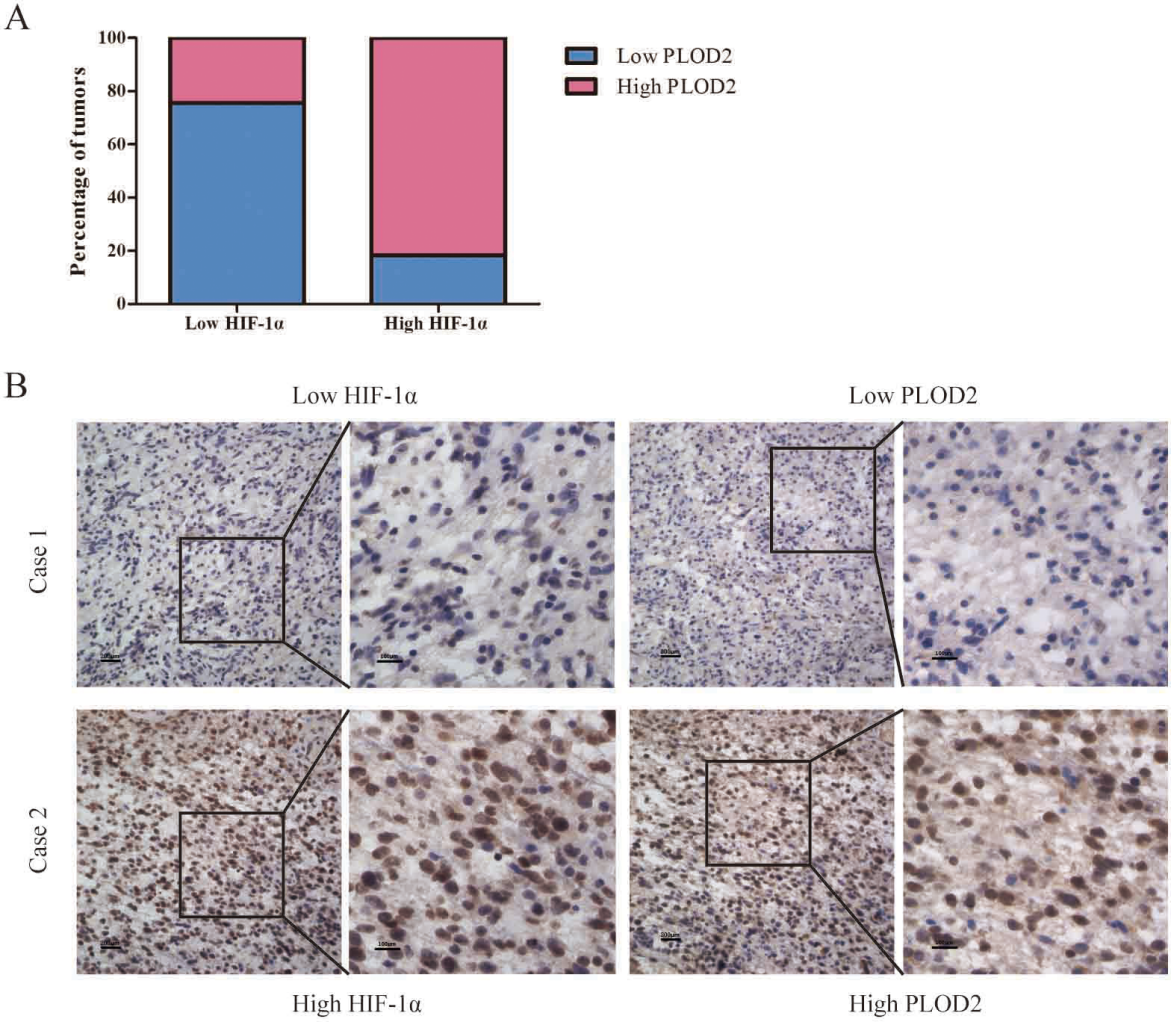
Association between PLOD2 and HIF-1α expression in clinical glioma samples (**A**) Percentage of glioma specimens with low or high PLOD2 expression in relation to the expression of HIF-1α. (**B**) Co-expression of PLOD2 and HIF-1α in surgical glioma specimens.

**Figure 9:**
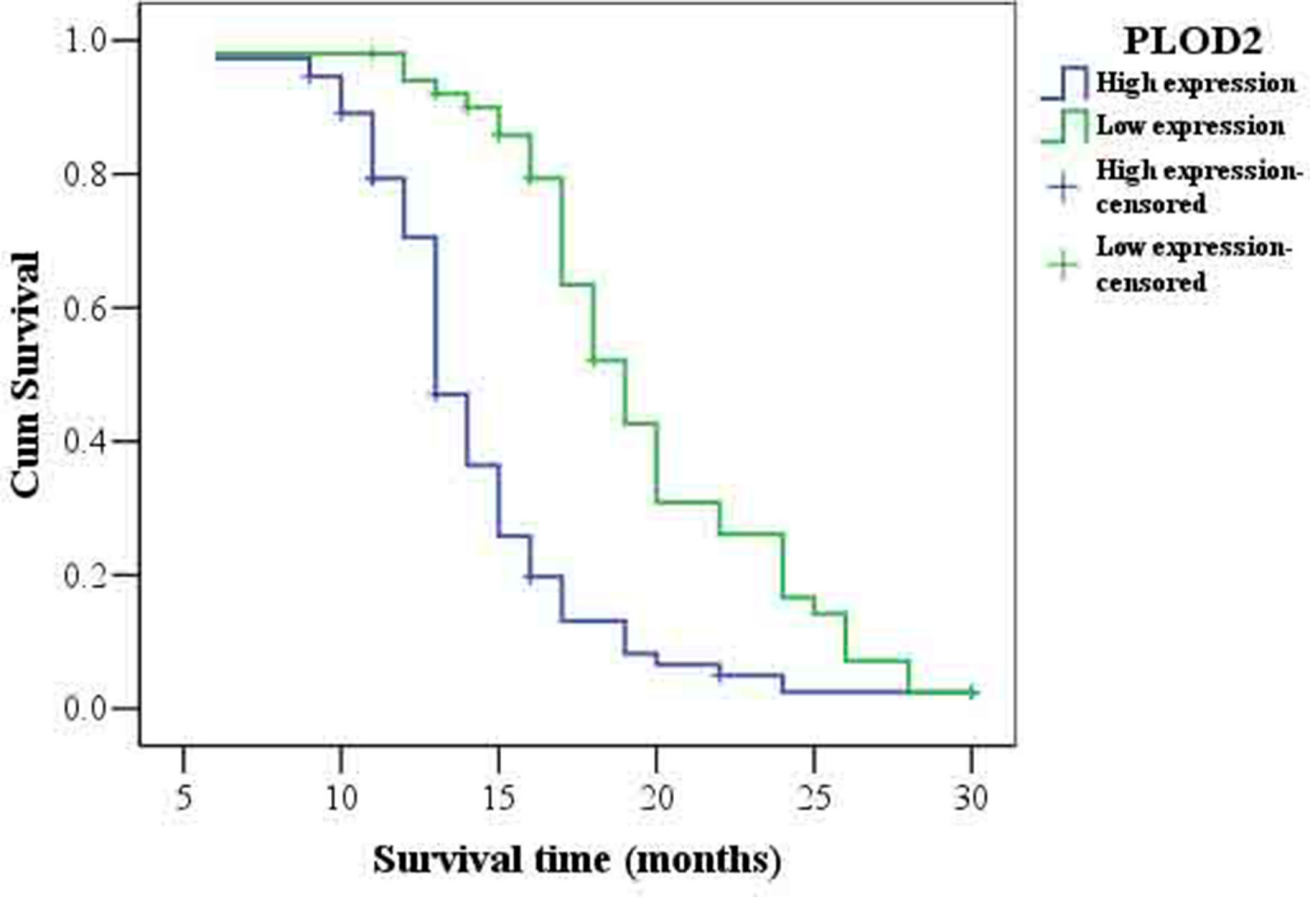
Kaplan-Meier survival analysis of overall survival in all patients with glioma according to PLOD2 protein expression Accumulation expression of PLOD2 was unfavorable for glioma prognosis. The log-rank test was used to calculate *P* values.

As summarized in Table 2, Chi-square test showed that the levels of PLOD2 protein significantly correlated with the status of pathology classification (WHO I–II vs. III–IV) in 125 glioma patients (*P* = 0.011). There was no significant association between PLOD2 expression and other characteristics. To further confirm the relationship between PLOD2 and HIF-1α in human glioma tissues, the levels of HIF-1α protein were evaluated in the same set of 125 glioma tissues by immunohistochemistry analysis. Prominent PLOD2 staining was found in 74 of 125 patients with glioma while prominent HIF-1α staining was found in 76 of 125 patients. In addition, Spearman's correlation analysis indicated a significant positive correlation between PLOD2 and HIF-1α expression levels (*R* = 0.567, *P* < 0.001, Figure 8, Table 3), consistent with our previous finding that HIF-1α increased PLOD2 expression *in vitro*. Furthermore, the follow-up data of the 125 glioma patients was used to access the prognosic value of PLOD2 for predicting patient survival. Kaplan-Meier survival analysis revealed that patients who had low PLOD2 levels had a significantly better outcome (*P* < 0.001) (Figure 9).

**Table 2:** Correlation between the clinicopathological factors and expression of PLOD2 in glioma

Factors	*N*	PLOD2 expression			
		High	Low	χ^2^	*P*
**Gender**					
**Male**	81	51 (63.0%)	30 (37.0%)	1.349	0.245
**Female**	44	23 (52.3%)	21 (47.7%)		
**Age**					
**≥ 50**	76	48 (63.2%)	28 (36.8%)	1.257	0.262
**< 50**	49	26 (53.1%)	23 (46.9%)		
**Histological type**					
**AT**	80	47 (58.8%)	33 (41.3%)	0.984	0.611
**OT**	19	13 (68.4%)	6 (31.6%)		
**Other**	26	14 (53.8%)	12 (46.2%)		
**WHO grade**					
**I + II**	36	15(41.7%)	21(58.3%)	6.435	0.011
**III + IV**	89	59(66.3%)	30(33.7%)		

**Table 3:** HIF-1 alpha and PLOD2 correlation analysis in glioma tissue samples

HIF-1α expression	PLOD2 expression		*N*	rs	*P*
	Low	High			
**Low**	37	12	49	0.567	< 0.001
**High**	14	62	76		
**Total**	51	74	125		

## DISCUSSION

Although altered PLOD2 expression was observed in glioblastoma [13], the biological functions of PLOD2 in glioma tumorigenesis and progression have not been well characterized. Here, PLOD2 was confirmed to be frequently up-regulated in glioma by qRT-PCR, western blot and immunohistochemistry analyses. Clinical data revealed that expression of PLOD2 was significantly correlated with the status of pathology classification in human glioma. Moreover, higher level of PLOD2 was associated with shorter overall survival in patients with glioma, indicating that PLOD2 may serve as a prognostic indicator for glioma. Consistent with our results, PLOD2 overexpression has been reported to be associated with the clinical progression and poor outcome of patients with bladder cancer, hepatocellular carcinoma or glioblastoma [14, 15]. Up-regulation of PLOD2 has also been found in colorectal cancer, cervical cancer and hepatocellular carcinoma [11, 12, 14, 15].

In this study, we investigated the biological functions of PLOD2 in the development and progression of glioma. Decreased expression of PLOD2 converted U87 and U251 cells into less aggressive cells, with lower capability of proliferation, migration and invasion *in vitro* and *in vivo*. This is the first report to demonstrate the functional significance of PLOD2 in glioma.

Although we detect the indirect interactions between PLOD2 and molecules related to proliferation, migration and invasion, the exact mechanism of these interactions is not clear. It remains for future investigation to unveil the mechanism.

It has been reported that PLOD2 is required for metastasis to lymph nodes and lungs in breast cancer [21]. Nevertheless, the exact molecular mechanisms on how tumor cell migration and invasive potential are affected remain largely unknown. EMT, which is characterized as the down-regulation of epithelial markers, particularly E-cadherin, and the upregulation of mesenchymal markers, particularly vimentin, N-cadherin, and several key EMT transcription factors, such as snail and slug, is a crucial step for cancer cell migration and invasion in various cancer types [24–26]. Therefore, to elucidate the precise mechanisms involved in PLOD2-induced cell migration and invasion, the effects of PLOD2 on EMT-associated proteins were examined. Here, we showed that the molecular mechanisms underlying PLOD2-regulated cell migration may be the induction of EMT, as suppressed expression of PLOD2 resulted in elevated expression of E-cadherin and reduced expression of vimentin, N-cadherin, snail and slug.

Previous study revealed that the PI3K/AKT pathway is a major cascade mediating human cancer proliferation, growth, survival and metastasis [27]. Once activated, PI3K/AKT induces the phosphorylation of GSK-3β and subsequently regulates the expression of β-catenin which plays an important role in various tumor growth, migration and invasion [28–30]. Moreover, activation of PI3K/AKT is reported to decrease the cellular levels of E-cadherin and induce the levels of snail, slug, vimentin and N-cadherin expression, thereby inducing EMT and promoting metastasis [31–33]. Here, our data revealed that knockdown of PLOD2 resulted in decreased level of p-PI3K, p-AKT, p-GSK-3β and β-catenin, implicating that inactivated PI3K/AKT signaling may be responsible for shPLOD2-mediated suppression of glioma cell proliferation, migration and invasion. The altered expression of E-cadherin, snail, slug, vimentin and N-cadherin with suppressed expression of PLOD2 probably resulted from inactivation of PI3K/AKT pathway. Taken together, our data suggested that inhibition of PLOD2 attenuates glioma cell proliferation, migration and invasion through modulating multiple EMT-associated factors via PI3K/AKT signaling.

Hypoxia is implicated in many aspects of glioma, such as invasion, apoptosis, chemoresistance, resistance to antiangiogenic therapy and radiation resistance [34, 35]. Plenty of studies have shown that PLOD2 can be induced by HIF-1α under hypoxia in various cell types [15, 36]. Interestingly, HIF-1α has been reported to bind to the HREs within the promoter of a wide range of target genes, such as lysyl oxidase, Bmi1, Twist, SIP1 and snail [37–39]. Therefore, we investigated whether HIF-1α could regulate PLOD2 expression in glioma cells. Our data confirmed that silenced expression of HIF-1α lad to markedly suppressed PLOD2 expression in glioma cells under hypoxia. Consistently, the immunohistochemistry analysis of 45 glioma tissues also showed a positive correlation between PLOD2 and HIF-1α. Gilkes, D. M. and his colleagues have reported that PLOD2 is essential for hypoxia-induced breast cancer metastasis [21]. However, little is known about the function and molecular mechanism of PLOD2 in hypoxia-induced glioma migration and invasion. Here, this study provides the first piece of evidence that PLOD2 is involved in hypoxia-induced EMT in glioma cells. Knockdown of PLOD2 partially inhibited hypoxia-mediated glioma cell migration and invasion. Mechanistically, inhibition of PLOD2 partially reversed hypoxia-modulated expression of several EMT-associated factors, including E-cadherin, β-catenin, N-cadherin, vimentin, snail and slug. Taken together, our results demonstrate that PLOD2 mediates hypoxia-induced EMT, at least in part in glioma cells.

In summary, we demonstrate for the first time that knockdown of endogenous PLOD2 suppresses glioma cell proliferation, migration and invasion through modulating multiple EMT-associated factors via inactivation of PI3K/AKT signaling. Moreover, we have shown that PLOD2 is induced by HIF-1α and then mediates hypoxia-induced EMT and migration in glioma cells. Most importantly, we have found that PLOD2 may be a valuable prognostic marker of glioma. Our study demonstrates that PLOD2 is a potential oncogene participating in glioma pathogenesis and PLOD2 suppression might be a potential therapeutic strategy for glioma.

## MATERIALS AND METHODS

### Cell culture

The human glioma cell lines U251, U87, U118MG, A172and SHG44 were purchased from the Chinese Academy of Sciences (Shanghai, China). Cells were maintained in Dulbecco's modified Eagle's medium (DMEM) (Hyclone, Logan, UT USA) supplemented with 10% fetal calf serum (Hyclone, Logan, UT USA). All cell lines were incubated in a humidified atmosphere of 5% CO_2_ at 37°C. For the exposure of cells to hypoxia, U251 and U87 cells were cultured in a hypoxic chamber (Hypoxia incubator chamber, Heraeus, Germany) containing 1% O2, 5% CO2, and 94% N2.

### Patient tissue specimens and ethics statement

A total of 125 paraffin-embedded glioma and 30 normal brain samples were obtained from the Nanfang Hospital of Southern Medical University, Guangzhou, China. These glioma cases were from 46 males and 24 females with age ranging from 11 to 68 years (median age, 41.7 years). For the use of these clinical materials for research purposes, prior written informed consents from all the patients and approval from the Ethics Committees of Nanfang Hospital of Guangdong Province were obtained. All specimens had confirmed pathological diagnosis and were classified according to the World Health Organization (WHO) criteria.

### RNA isolation, reverse transcription, and qRT-PCR

Total RNA was extracted from U251 and U87 cell lines, glioma tissues and normal brain tissues using RNAiso Plus (Takara, Shiga, Japan). For PLOD2, RNA was transcribed into cDNA and amplified with specific sense: 5′- GCGTTCTCTTCGTCCTCATC-3′; antisense primer: 5′- GTGTGAGTCTCCCAGGATGC-3′. The ARF5(sense primer: 5′- ATCTGTTTCACAGTCTGGGACG -3′ and antisense primer: 5′- CCTGCTTGTTGGCAAATACC -3′) gene was used as an internal control to normalize PLOD2 expression. The assays were performed in accordance with manufacturer's instructions (Takara, Shiga, Japan). Specificity of amplification products was confirmed by melting curve analysis. PCR reactions for each gene were repeated three times. Independent experiments were done in triplicate.

### Immunohistochemistry

Paraffin sections (4 mm) were deparaffinized in 100% xylene and re-hydrated in descending ethanol series and water according to standard protocols. Heat-induced antigen retrieval was performed in 10 mM citrate buffer for 2 min at 100°C. Endogenous peroxidase activity and non-specific antigens were blocked with peroxidase blocking reagent containing 3% hydrogen peroxide and serum. The sections were then incubated with primary antibodies, including HIF-1 α (1:50; Novus Biologicals, USA), PLOD2 (1:100;Proteintech, USA), E-cadherin (1:100;Proteintech, USA), β-catenin (1:100; Proteintech, USA), and snail (1:100; Abcam, USA), at 4°C overnight. After washing, the sections were followed by incubation with biotin-labeled rabbit anti-goat antibody for 15 min at room temperature, and subsequently were incubated with streptavidin-conjugated horseradish peroxidase (HRP) (Maixin, Fuzhou, China). The peroxidase reaction was developed using 3,3-diaminobenzidine (DAB) chromogen solution in DAB buffer substrate. Sections were visualized with DAB and counterstained with hematoxylin, mounted in neutral gum, and analyzed using a bright field microscope equipped with a digital camera (Nikon, Japan).

### Evaluation of staining

The immunohistochemical stained tissue sections were reviewed and scored separately by two pathologists blinded to the clinical parameters. For cytoplasmic staining, the score was evaluated according to the sum of cytoplasm staining intensity and the percentage of positive staining areas of cells. The staining intensity was scored as previously described [40] 0–3 and the percentage of positive staining areas of cells was defined as a scale of 0–3 (0: < 10%, 1: 10–25%, 2: 26–75%, and 3: > 76%). For nuclear staining, the staining score was defined based on the sum of nuclear staining intensity and the number of positive nuclear staining. Nuclear staining intensity score was consistent with cytoplasm. The positive nuclear staining scores were defined as follows: 0: < 20%, 1: 20–49%, 2: 50–79%, and 3: > 80%. The sum of the cytoplasm and nuclear staining scores were used as the final staining score (0–12). For statistical analysis, a final staining score of 0–4 and 5–6 in cytoplasm or 0–3 and 4–6 in nucleus was considered to be low or high expression, respectively.

### Western blot analysis

Western blot was carried out according as described [41, 42] with rabbit polyclonal PLOD2, E-cadherin and β-catenin antibodies (1:1000; Proteintech, USA), PI3K, p-PI3K (Tyr458), AKT, p-AKT (Ser473), GSK-3β, p-GSK-3β, N-Cadherin, Slug and Vimentin antibodies (1:1000; Cell Signaling Technology, Danvers, MA, USA), as well as Snail antibody (1:1000; Abcam, USA). Mouse monoclonal HIF-1α antibody (1:50; Novus Biologicals, USA) was used for normalization. An HRP-conjugated anti-rabbit or anti-mouse IgG antibody was used as the secondary antibody (1:2000; CoWin Bioscience, Beijing, China). Signals were detected using enhanced chemiluminescence reagents (Pierce, Rockford, IL, USA).

### Establishment of glioma cell line with stable expression of PLOD2 short hairpin RNA

The preparation of lentiviruses expressing human PLOD2 short hairpin RNA (shRNA-2 3284-1,23285-1,23286-1) (Supplementary Table 1) were performed using the pLVTHM-GFP lentiviral RNAi expression system. U87 and U251 cells were infected with lentiviral particles containing specific or negative control vectors, and polyclonal cells with GFP signals were selected for further experiments using FACS flow cytometry.

### Transient transfection with siRNAs

Small-interfering RNA (siRNA) for PLOD2 and HIF-1α was designed and synthesized by Guangzhou RiboBio (RiboBio Inc, China). The sequence of each gene and their controls are shown in Supplementary Table 2. Three siRNAs targeting on PLOD2 gene were designed and synthesized, the most effective siRNA (siPLOD2) identified by Real Time-PCR was applied for the further experiments. Twenty-four hours prior to transfection, glioma cells U87 and U251 were plated onto a 6-well plate or a 96-well plate (Nest Biotech, China) at 30–50% confluence. Constructs were then transfected into cells using TurboFect TM siRNA Transfection Reagent (Fermentas, Vilnius, Lithuania) according to the manufacturer's protocol. Cells were collected after 48–72 h for further experiments.

### Wound-healing assay

Cell migration was assessed using scratch-healing assays. Briefly, U251 and U87 cells stably or transiently transfected with shRNA or siRNA and empty vectors were cultured in 6-well plates. When the cells grew to 90% confluence, three scratch wounds across each well were made using a P-200 pipette tip. Fresh medium supplemented with reduced (5%) fetal bovine serum was added, and the wound-closing procedure was observed for 48 h. Photographs were taken at 0 and 24 h, respectively.

### Cell proliferation assays

The MTT assay was used to examine cell proliferation rate. Glioma cells (1,000/well) were seeded in 96-well plates with a volume of 200ul medium. Subsequently, 20 ul of MTT (5 mg/ml in PBS) (Sigma, St Louis, MO, USA) solution was added to each well and incubated for 4 h. The formazan crystals formed by viable cells were solubilized in 150 ml dimethyl sulfoxide (Sigma, St Louis, MO, USA) and then the absorbance value (OD) was measured at 490 nm. The observation duration lasts for a week.

### Cell migration and invasion assays

*In vitro* cell migration and invasion assays were examined according to our previous study [43]. For the cell migration assay, 1 × 10^4^ cells in 100 μl DMEM medium without FBS were seeded on a fibronectin-coated polycarbonate membrane insert in a Transwell apparatus (Costar, MA). In the lower chamber, 500 ul DMEM with 10% FBS was added as chemoattractant. After the cells were incubated for 6 hours at 37°C in a 5% CO_2_ atmosphere, the insert was washed with PBS, and cells on the top surface of the insert were removed with a cotton swab. Cells adhering to the lower surface were fixed with methanol, stained with crystal violet solution and counted under a microscope in five predetermined fields (200×). All assays were independently repeated at least thrice. For the cell invasion assay, the procedure was similar to the cell migration assay, except that the transwell membranes were precoated with 24 μg/μl Matrigel (R&D Systems, USA) and the cells were incubated for 8 hours at 37°C in a 5% CO_2_ atmosphere. Cells adhering to the lower surface were counted the same way as the cell migration assay.

### Intracranial injection model

Female mice (4–6 weeks old) were obtained from SUN YAT-SEN University (Guangzhou, China). They were acclimatized for 1–2 weeks. Mice were anesthetized with 1% pentobarbital sodium and placed in a stereotactic frame (RWD Life Science, Shenzhen, China) prior to the inoculation of cells. A total of 1 × 10^6^ U87 cells transfected with shPLOD2 or mock U87 cells (*N* = 5 per group) were suspended in 10 ul PBS and injected into the left hemisphere using a 10 ul syringe and needle (Shanghai Jiaan analyzer factory, Shanghai, China). The injection point was located 1 mm posterior of bregma, 2 mm left of the midline. Mice were sacrificed and the brains were harvested for further HE-staining after 40 days of inoculation.

### Statistical analysis

All quantified data represented an average of at least triplicate samples. SPSS 13.0 and Graph Pad Prism 5.0 software were used for statistical analysis. Data are presented as mean ± SD. One-way ANOVA or two-tailed Student's *t-test* was used for comparisons between groups. Chi-square test or Fischer's was used to identify the differences between categorical variables. Partial correlations were applied in multivariate correlations analysis. Survival analysis was performed using Kaplan-Meier method. Multivariate Cox proportional hazards method was used for analyzing the relationship between the variables and patient's survival time. Differences were considered statistically significant when *P* < 0.05. Data shown is mean ± SEM unless indicated otherwise.

## SUPPLEMENTARY TABLES AND FIGURES



## Abbreviations

PLOD2: procollagen-lysine, 2-oxoglutarate 5-dioxygenase 2
HIF-1α: hypoxia-inducible factor-1α
HREs: hypoxia response elements
EMT: epithelial–mesenchymal transition
PI3K: phosphoinositide 3-kinase
GSK-3β: glycogen synthase kinase-3β
qRT-PCR: quantitative real-time polymerase chain reaction
siRNA: small interfering RNA
