# Supplementary material for: Hypoxia-induced PLOD2 promotes proliferation, migration and invasion via PI3K/Akt signaling in glioma
Source: Oncotarget. 2017 Mar 30;8(26):41947–62. doi: 10.18632/oncotarget.16710 (PMC5522040; doi:10.18632/oncotarget.16710)
Supplement: Supplementary file 1 [file oncotarget-08-41947-s001.pdf]

Hypoxia-induced PLOD2 promotes proliferation, migration and invasion via PI3K/Akt signaling in glioma

Supplementary Materials

Supplementary Table 1: The sequence of shPLOD2

NO.	5′	STEM	Loop	STEM	3′
PLOD2-RNAi-1-a	Ccgg	ggAAATGGACCCACCAAGATT	CTCGAG	AATCTTGGTGGGTCCATTTCC	TTTTTg
PLOD2-RNAi-1-b	aattcaaaaa	ggAAATGGACCCACCAAGATT	CTCGAG	AATCTTGGTGGGTCCATTTCC	
PLOD2-RNAi-2-a	Ccgg	ctTTGCCGAAATGCTAGAGAA	CTCGAG	TTCTCTAGCATTTCGGCAAAG	TTTTTg
PLOD2-RNAi-2-b	aattcaaaaa	ctTTGCCGAAATGCTAGAGAA	CTCGAG	TTCTCTAGCATTTCGGCAAAG	
PLOD2-RNAi-3-a	Ccgg	gtCATATTTGCTGGTGGTCCA	CTCGAG	TGGACCACCAGCAAATATGAC	TTTTTg
PLOD2-RNAi-3-b	aattcaaaaa	gtCATATTTGCTGGTGGTCCA	CTCGAG	TGGACCACCAGCAAATATGAC	

Supplementary Table 2: The sequence of siPLOD2

No	Sequence
siRNA-001	sense: 5′- GCUCCAUAUGUCAACCGUA dTdT-3′;<br>anti-sense: 3′- dTdT CGAGGUAUACAGUUGGCAU-5′
siRNA-002	sense: 5′- GCAGUAGAUGUCCAUCCAA dTdT-3′;<br>anti-sense: 3′- dTdT CGUCAUCUACAGGUAGGUU-5′
siRNA-002	sense: 5′- GGAGUUCAUUGCACCAGUU dTdT-3′;<br>anti-sense: 3′- dTdT CCUCAAGUAACGUGGUCAA-5′

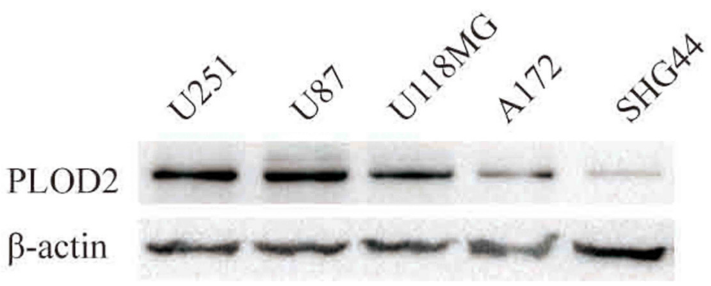

Supplementary Figure 1: The expression levels of PLOD2 protein were assessed in different glioma cell lines by western blot. β-actin was used as a loading control.

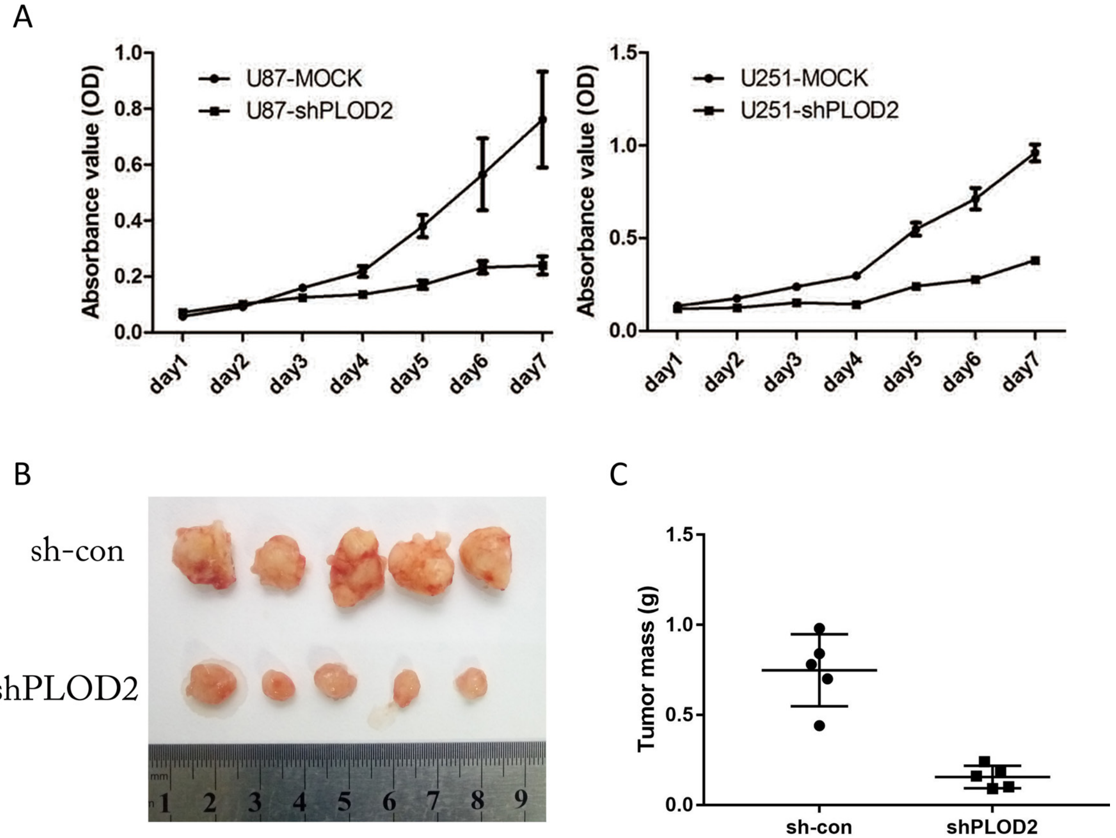

Supplementary Figure 2: (**A**) Effect of shPLOD2 on U251 and U87 cell proliferation compared to untreated cells was measured by MTT assay. Absorbance was read at 490 nm with averages from triplicate wells. Data are presented as mean ± SD. (**B**) Image of tumors that developed in xenograft-transplanted nude mouse tumor models 5 weeks after subcutaneous injection of sh-PLOD2 or sh-con U87 cells. (**C**) Weights of xenografts established by subcutaneous transplantation with sh-PLOD2 or sh-con U87 cells 5 weeks after cell injection.

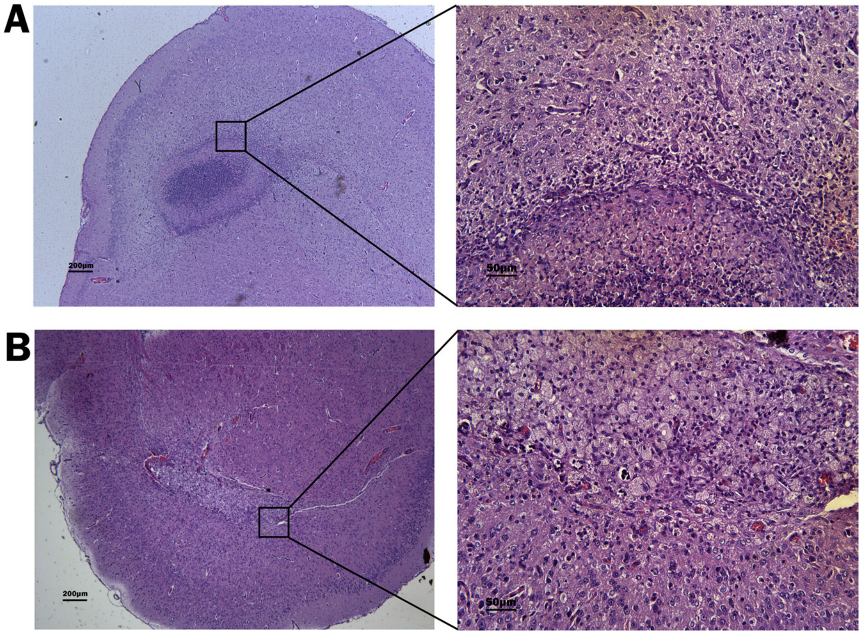

Supplementary Figure 3: shPLOD2 transfected U87 exhibited attenuated potential of invasion *in vivo*. Representative H&E staining of U87-MOCK (**A**) and U87-shPLOD2 intracranial xenograft (**B**). Magnification in left panels is 4×, scale bar is 200 μm; magnification in right panels is 20×, scale bar is 50 μm.

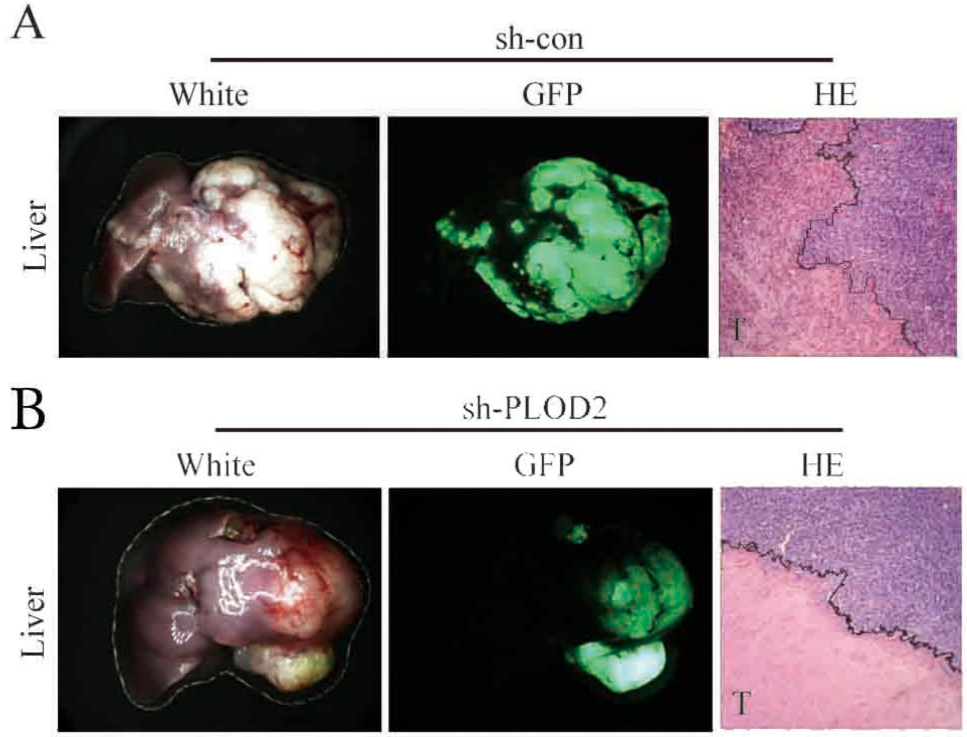

Supplementary Figure 4: Knockdown of PLOD2 inhibits the invasive potential of glioma *in vivo*. External fluorescence images of liver were obtained 40 days after spleen injection with sh-con (**A**) or sh-PLOD2 U87 (**B**) cells, respectively. H&E staining of tumor nodules in liver were shown. The boundary between tumor and normal tissue was indicated by black line (magnification, ×200).
